# Reinfection by the SARS-CoV-2 Gamma variant in blood donors in Manaus, Brazil

**DOI:** 10.1186/s12879-022-07094-y

**Published:** 2022-02-05

**Authors:** Carlos A. Prete, Lewis F. Buss, Renata Buccheri, Claudia M. M. Abrahim, Tassila Salomon, Myuki A. E. Crispim, Marcio K. Oikawa, Eduard Grebe, Allyson G. da Costa, Nelson A. Fraiji, Maria do P. S. S. Carvalho, Charles Whittaker, Neal Alexander, Nuno R. Faria, Christopher Dye, Vítor H. Nascimento, Michael P. Busch, Ester Cerdeira Sabino

**Affiliations:** 1 Department of Electronic Systems Engineering, University of São Paulo, Av. Professor Luciano Gualberto, Travessa 3, São Paulo, 158 Brazil; 2 Departamento de Moléstias Infecciosas e Parasitarias, Instituto de Medicina Tropical da Faculdade de Medicina da Universidade de São Paulo, Av Dr Eneas de Carvalho 470, 1º andar, São Paulo, 05403-000 Brazil; 3 Vitalant Research Institute, 270 Masonic Avenue, San Francisco, CA USA; 4 Fundação Hospitalar de Hematologia e Hemoterapia do Amazonas, Av. Constantino Nery, Manaus, 4397 Brazil; 5 Faculdade de Ciências Médicas de Minas Gerais, Alameda Ezequiel Dias, Belo Horizonte, 275 Brazil; 6 Center of Mathematics, Computing and Cognition, Universidade Federal do ABC, Rua Arcturus, 03, São Bernardo do Campo, Brasil; 7 University of California San Francisco, 1001 Potrero Ave, San Francisco, CA USA; 8 SACEMA, Stellenbosch University, 19 Jonkershoek Rd, Stellenbosch, South Africa; 9 MRC Centre for Global Infectious Disease Analysis, and the Abdul Latif Jameel Institute for Disease and Emergency Analytics (J-IDEA), School of Public Health, Imperial College London, South Kensington Campus, London, SW7 2AZ UK; 10 Department of Infectious Disease Epidemiology, London School of Hygiene & Tropical Medicine, LSHTM, Keppel Street, London, WC1E 7HT UK; 11 Department of Zoology, University of Oxford, South Parks Road, Oxford, OX1 3SZ UK

**Keywords:** COVID-19, SARS-CoV-2, Gamma, P.1, Reinfections, Blood donors, Herd immunity, Manaus, Amazon, Brazil

## Abstract

**Background:**

The city of Manaus, north Brazil, was stricken by a second epidemic wave of SARS-CoV-2 despite high seroprevalence estimates, coinciding with the emergence of the Gamma (P.1) variant. Reinfections were postulated as a partial explanation for the second surge. However, accurate calculation of reinfection rates is difficult when stringent criteria as two time-separated RT-PCR tests and/or genome sequencing are required. To estimate the proportion of reinfections caused by Gamma during the second wave in Manaus and the protection conferred by previous infection, we identified anti-SARS-CoV-2 antibody boosting in repeat blood donors as a mean to infer reinfection.

**Methods:**

We tested serial blood samples from unvaccinated repeat blood donors in Manaus for the presence of anti-SARS-CoV-2 IgG antibodies using two assays that display waning in early convalescence, enabling the detection of reinfection-induced boosting. Donors were required to have three or more donations, being at least one during each epidemic wave. We propose a strict serological definition of reinfection (reactivity boosting following waning like a V-shaped curve in both assays or three spaced boostings), probable (two separate boosting events) and possible (reinfection detected by only one assay) reinfections. The serial samples were used to divide donors into six groups defined based on the inferred sequence of infection and reinfection with non-Gamma and Gamma variants.

**Results:**

From 3655 repeat blood donors, 238 met all inclusion criteria, and 223 had enough residual sample volume to perform both serological assays. We found 13.6% (95% CI 7.0–24.5%) of all presumed Gamma infections that were observed in 2021 were reinfections. If we also include cases of probable or possible reinfections, these percentages increase respectively to 22.7% (95% CI 14.3–34.2%) and 39.3% (95% CI 29.5–50.0%). Previous infection conferred a protection against reinfection of 85.3% (95% CI 71.3–92.7%), decreasing to respectively 72.5% (95% CI 54.7–83.6%) and 39.5% (95% CI 14.1–57.8%) if probable and possible reinfections are included.

**Conclusions:**

Reinfection by Gamma is common and may play a significant role in epidemics where Gamma is prevalent, highlighting the continued threat variants of concern pose even to settings previously hit by substantial epidemics.

**Supplementary Information:**

The online version contains supplementary material available at 10.1186/s12879-022-07094-y.

## Introduction

Approximately 76% of the inhabitants of Manaus had been infected with SARS-CoV-2 eight months after the first reported case in March 2020 [1]. Nevertheless, a second epidemic wave occurred in the city, coinciding with the emergence of a new SARS-CoV-2 variant of concern (VOC) in November 2020 first denoted P.1, and recently classified as the Gamma variant of concern by WHO [2]. This variant corresponded to 87% of all infections in January 2021 [3].

Both increased transmissibility and the ability to partially evade protective immunity have been postulated to explain the Gamma-driven resurgence of COVID-19 in Manaus [3, 4]. Whilst a significant body of work supports increases to transmissibility of both Gamma and other variants of concern [5–7], comparatively little work has explored the potential for reinfection by these lineages, despite significant in vitro evidence supporting partial immune-escape [8]. It is therefore essential to understand the rate of reinfection in order to predict how this variant (and others with immune-escape potential) will spread through Brazil and other regions of the globe that have experienced significant previous outbreaks and are at risk for reinfections.

Individual cases of reinfection by the Gamma variant have been widely reported in the literature [9–11], but the frequency of these cases at a population-level has not been established. Detecting reinfections directly by testing of swabs from recurrent symptomatic infections is difficult because most SARS-CoV-2 infections are undiagnosed asymptomatic or mild cases [12], leading to a small number of patients with two confirmed infections. Instead, we assess the reinfection rate using samples of repeat blood donors, allowing the detection of asymptomatic infections.

## Methods

We retrieved and tested serial samples from unvaccinated repeat donors from Manaus with three or more donations, which included at least one during the first epidemic wave (between April 1st and June 30th, 2020) and at least one after January 1st, 2021. These criteria select donors solely based on the donation date, thus selected donors and the set of all repeat blood donors have a similar proportion of infection. We excluded donors that had their first anti-N positive result in November and December 2020, when it was not possible to determine whether the infection was caused by Gamma due to its low prevalence at that time. Given this exclusion, no infections observed in selected donors in 2020 are due to Gamma because this VOC had an insignificant prevalence before November 2020 [3].

The samples were first tested using an anti-N SARS-CoV-2 IgG chemiluminescence microparticle assay (CIMA, Abbott Park, IL, USA), and then the samples with enough volume were retested using the Abbott anti-S SARS-CoV-2 IgG CIMA. These are high specificity assays whose reactivity consistently wanes during convalescence [1, 13, 14] and presents small measurement error (see Additional file 1: Appendix). Furthermore, because the anti-S assay shows smaller reactivity waning than the anti-N assay, it may be able to detect infections that remained undetected by the anti-N assay.

In order to detect reinfections, we hypothesized that reinfection would induce anamnestic “boosting” of plasma anti-N and anti-S IgG antibody levels, yielding a V-shaped time series of antibody reactivity levels. We also assumed that a V-shaped antibody curve can only be caused by reinfection, since exposure should only lead to an increase of the antibody level if there is significant viral replication, hence an infection. This strict serological definition of reinfection is only valid for assays that show consistent reactivity waning over time, since reinfections may not produce a V-shaped curve in assays with no reactivity waning.

Repeat donors were partitioned into six groups that reflect the inferred sequence of infection and reinfection with non-Gamma and Gamma variant. To define these groups, we classified donors based on the serial anti-N samples obtaining an assay-specific classification, and repeated the procedure for the serial anti-S samples. As such, each donor was assigned to two assay-specific groups (one for each assay), which were then combined according to Table 1 to obtain the final classifications. Donors with insufficient sample volume for testing with the anti-S assay were classified as “unknown”. Reinfections detected by only one assay were classified as “possible reinfections”, and only cases of reinfections detected by both assays had their final classification assigned as “reinfection”. Therefore, the rules for obtaining the final groups are conservative.

**Table 1:** Final classification for each donor based on the assay-specific classifications obtained with the anti-N and anti-S assays, and the number of donors assigned to each group

Assay-specific classification of donors (anti-S assay)						
	Persistently seronegative	Infection by non-Gamma variant	Infection by Gamma	Reinfection by Gamma	Probable reinfection by Gamma	Unknown
Assay-specific classification of donors (anti-N assay)						
Persistently seronegative	Persistently seronegative (60)	Infected by non-Gamma variant (4)	Infected by Gamma (3)	Possible reinfection by Gamma (1)		Persistently seronegative (4)
Infection by non-Gamma variant		Infected by non-Gamma variant (77)		Possible reinfection by Gamma (14)	Infected by non-Gamma variant (2)	Infected by non-Gamma variant (7)
Infection by Gamma	Infected by Gamma (1)		Infected by Gamma (43)	Possible reinfection by Gamma (1)		Infected by Gamma (4)
Reinfection by Gamma	Possible reinfection by Gamma (1)		Possible reinfection by Gamma (1)	Reinfection by Gamma (8)		
Probable reinfection by Gamma					Probable reinfection by Gamma (7)	

Empty cells represent groups with no donors. Text within the cells denotes the final classification assigned to each case

To design the inclusion rules for each group, we assumed that all positive cases in 2021 are due to Gamma because of its high prevalence (87% of sequenced samples) in early January, which likely increased in the following months due to the higher transmissibility compared to non-Gamma variants circulating in Manaus[3]. Additional file 1: Fig. S1 shows a flowchart illustrating the classification rules, and Additional file 1: Fig. S2 describes an illustration of the procedure used to classify donors. The groups and their corresponding definitions are listed below, and are also summarized in Table 2.

(A) Persistently seronegative
  Donors that never had a positive test result. It is not possible to say that all persistently seronegative donors were not infected, since some infected donors may have had already seroreverted at the date of sample collection, or not seroconverted at all.
(B) Infection by non-Gamma variant
  Two requirements are needed for a donor to be included in this group. First, the donor must have a positive test result before November 1^st^, 2020. Since donors that had their first positive result in November and December 2020 were excluded, this requirement is equivalent to requiring a positive donation in 2020 and a negative donation in 2021. Donors must also fill one of the following rules:
  - All sample test results in 2021 are negative.
  - There are positive donations in 2021, but none of them have a rising result.
(C) Infection by Gamma
  Donors that did not have any positive test result in 2020 and some positive result in 2021. Some of these cases may be unobserved reinfections by Gamma in the case of an undetected infection in 2020.
(D) Reinfection by Gamma
  A donor is classified as a case of reinfection by Gamma when filling some of the following conditions:
  - Donors with a positive donation in 2020 and another positive donation in 2021 with a V-shaped curve ending in 2021. In other words, these are donors that have a positive donation in 2020, a second donation with lower S/C value (that could be positive or negative), followed by a positive donation in 2021 with an increase in S/C value. These donors were seroreverting and then seroconverted again due to the reinfection.
  - Donors with three consecutive rising positive results, the last being in 2021. Since the minimum interval between successive donations is 60 days for men and 90 days for women in Brazil, donors with three consecutive rising positive results would apparently be seroconverting for more than 120 days. Since this long seroconversion period is unlikely, $\mathrm{d}$onors following this rule have likely had an unobserved S/C decay after the second rising result, seroconverting again after being reinfected.
(E) Probable reinfection by Gamma
  Donors with two consecutive rising positive test results, the last being in 2021, separated by an interval $\Delta t\ge\Delta {t}_{\mathrm{min}}$, where $\Delta {t}_{\mathrm{min}}$ is a predefined parameter equal to 141 days and 126 days for the anti-N and anti-S assays, respectively (see Additional file 1: Appendix for an explanation on how $\Delta {t}_{\mathrm{min}}$ was obtained). We hypothesize that donors following this rule have had an unobserved antibody decline after the first positive sample, and seroconverted again after being reinfected. A minimum interval between donations is required to avoid misclassifying donors sampled during the seroconversion period as probable reinfections.

**Table 2:** Summarized definition and size of the groups used to classify donors for each assay

Infection group	Definition	Number of donors		
		Assay-specific classification (anti-N)	Assay-specific classification (anti-S)	Final classification
Persistently seronegative	No positive results	72	62	64
Infection by non-Gamma variant	A positive result before Nov 1st 2020 and decaying antibody levels in 2021	100	81	90
Infection by Gamma	No positive results in 2020 and a positive result in 2021	49	47	51
Reinfection by Gamma	A positive result in 2021 and before Nov 1st 2020 and an intermediate result with value below these two readings (V-shaped S/C time series)	9	22	8
	A positive result in 2021 succeeding two consecutive rising positive results in 2020$.$ Donors in this group have an observed seroconversion period much longer than expected	1	2	0
Probable reinfection by Gamma	One positive result in 2020 and a higher positive result in 2021 separated by an interval of at least $\Delta {t}_{\mathrm{min}}$ (seroconversion period longer than expected)	7	9	7
Possible reinfection by Gamma	Classification as “Reinfection by Gamma” by only one assay (reinfection detected by one assay but not both)			18
Unknown (anti-S assay only)	Not enough volume to retest the sample with the anti-S assay		15	
Total	.	238	238	238

The final classification was obtained by combining the groups assigned by both assays according to Table 1. The definitions of probable reinfections depend on the parameter $\Delta {t}_{\mathrm{min}}=141$ days for the anti-N assay and 126 days for the anti-S assay

**Fig. 1:**
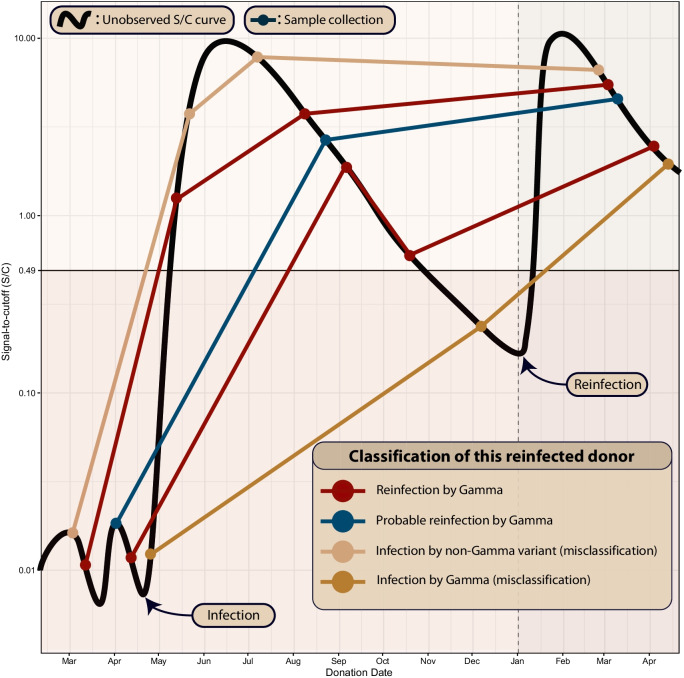
Illustration of an idealized signal-to-cutoff (S/C) curve of a reinfected individual that is assigned to different groups depending on the sequence of dates of sample collection. The black curve represents the unobserved trajectory of S/C over time, and circles represent sample collections. This figure shows five sets of serial samples that were collected in different dates. The patterns that can be confidently attributed to reinfection are shown in red: sampled points that reveal the underlying V-shaped curve, or three consecutive rising values that can only be obtained by sampling the underlying V-shaped curve. If the dates of sample collection are too sparse, this reinfection individual may be misclassified as “Infection by non-Gamma variant” or “Infection by Gamma”

These rules imply that a truly reinfected individual may be misclassified as “Infection by Gamma” or “Infection by non-Gamma variant” if samples are not collected shortly after the first infection, underestimating the proportion of reinfections. To partially account for this problem, we define probable reinfections (two consecutive reactivity boostings that are spaced by a minimum interval $\Delta {t}_{\mathrm{min}}$ larger than the expected seroconversion period, that is, a reinfection not sampled frequently enough to yield a V-shaped curve) and possible reinfections (reinfections detected by only one assay). The effect of misclassification due to sparse sampling is illustrated in Fig. 1, which shows the idealized signal-to-cutoff curve of a reinfected individual and the corresponding classification based on different possibilities for the sequence of dates of sample collection.

Information on symptom severity or hospitalization of infected donors was not available. However, the cohort of selected repeat blood donors is likely biased towards non-hospitalized individuals with mild or asymptomatic infection because symptomatic donors are unable to donate for 30 days after the end of symptoms. Also, few selected donors were hospitalized in the first wave because they returned to donate multiple times over the course of the year.

## Results

**Fig. 2:**
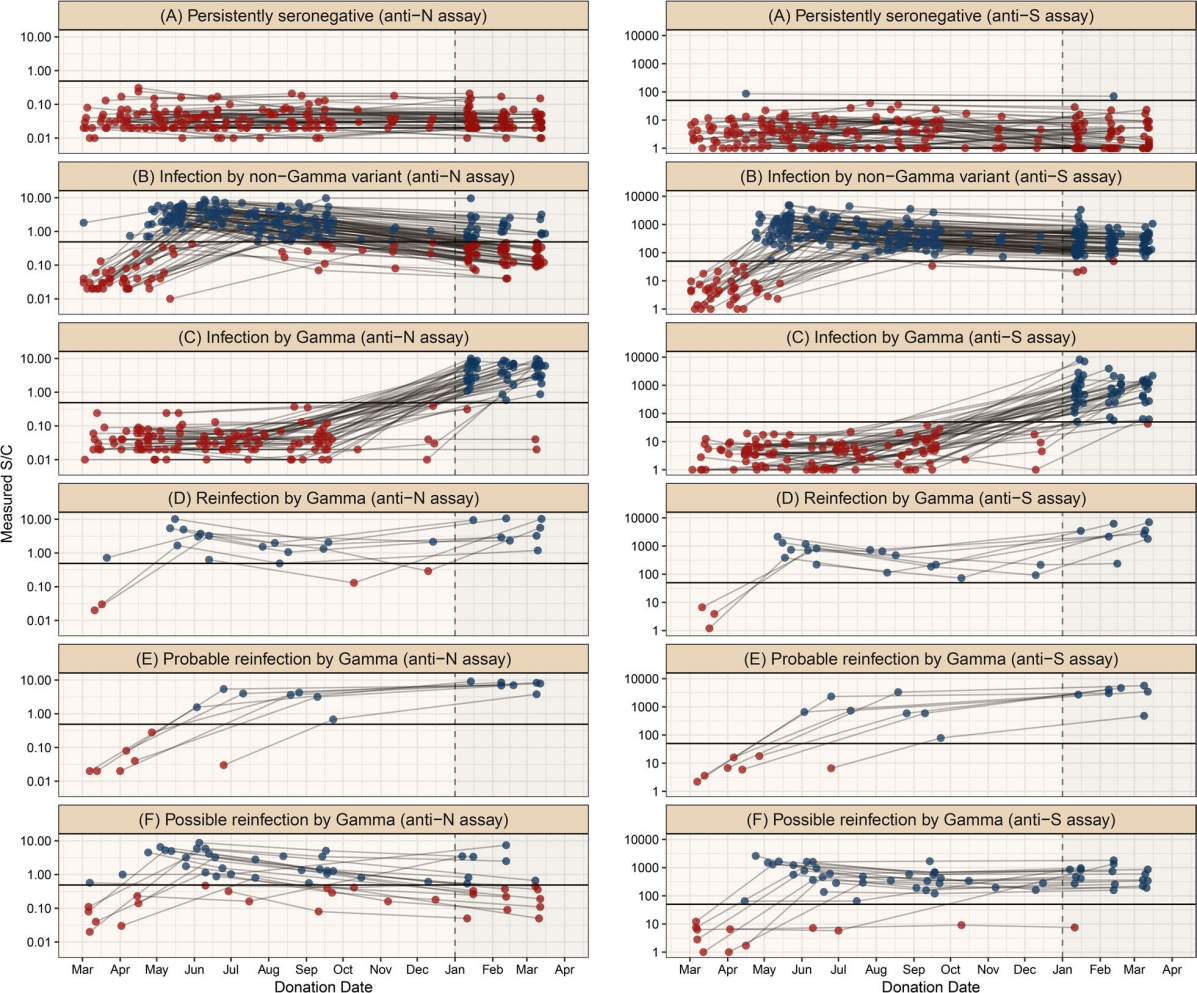
Classification of the repeat blood donors according to their antibody profile. Each facet shows the serial results obtained with the anti-N or anti-S IgG assays for donors in the corresponding group. Blue and red dots represent respectively positive and negative results, and donations from the same donor are connected by a line. Because 18 samples could not be retested with the anti-S assay, less than three anti-S results are shown for some donors, which were classified based solely on the serial anti-N results

During the study period, we identified 3655 repeat blood donors, of which 782 donated three or more times, and 240 met all our inclusion criteria (see Additional file 1: Fig. S1). Two donors were excluded for having their first anti-N positive result in November or December 2020 (when it is not possible to identify if infection was caused by Gamma), resulting in 238 donors selected for this study. The median (IQR) age was 36.5 (28.0–44.0) and 12.8% were female. 85.4% of donors self-declared as *pardo* (mixed ethnic ancestries), 11.1% as white and 3.0% as black, compared with 75.7%, 20.7% and 2.9% for the estimated population in Manaus self-declared as *pardo*, white and black individuals (see Additional file 1: Fig. S5). There were 18 samples tested only with the anti-N assay because they did not have enough volume to be tested for the second assay. For this reason, only 223 donors were classified based on the anti-S assay patterns over time, and the remaining 15 donors were classified based solely on the anti-N assay. Figure 2 presents the serial results of all 238 selected donors, and Table 1 shows the number of donors assigned to each assay-specific group and their corresponding final classification. The assays showed a high concordance, with 87.4% of donors receiving the same classification on both assays. Table 2 summarizes the definition of groups and contains the sizes of the assay-specific and final groups, and the serial results of each assay-specific group for both assays are shown in Additional file 1: Figs. S3, S4.

There were 59 presumed Gamma infections in 2021, of which 8 (13.6%, 95% CI 7.0–24.5%) had a V-shaped curve indicating reinfection by both anti-N and anti-S assays. The anti-S assay detected 16 cases of reinfection that were undetected by the anti-N assay; these were given a final classification of possible reinfection. If probable and possible reinfections are included, these percentages increase to 22.7% (95% CI 14.3–34.2%), or 39.3% (95% CI 29.5–50.0%), respectively. These eight Gamma reinfections also represent 6.5% (95% CI 3.3–12.3%) of the 123 individuals that had a primary infection in the first wave, increasing to 12.2% (95% CI 7.5–19.1%) and 26.8% (95% CI 19.8–35.3%) if probable and possible reinfections are considered.

Of 115 previously negative individuals, 51 (44.3%, 95% CI 35.6–53.5%) were infected by Gamma over the time-period considered. As such, the protection against reinfection conferred by previous infection (defined as 100 × [1 – relative risk of reinfection]) is 85.3% (95% CI 71.3–92.7%), or 72.5% (95% CI 54.7–83.6%) and 39.5% (95% CI 14.1–57.8%) if probable or possible reinfections are included.

## Discussion

The high proportion of Gamma reinfections suggests that reinfection with this variant is common and may play a significant role in Gamma-prevalent regions. The estimated relative risk of reinfection shows that even though a previous infection decreases the chance of reinfection, the protection is not close to 100%. Hence, immunity against non-Gamma variants achieved by natural infection may not prevent new outbreaks caused by Gamma. This conclusion may also extend to other variants where in vitro results support immune-escape potential equal or larger than Gamma.

Our proportion of reinfections by Gamma is compatible with a previous estimate of 28% obtained from a compartmental model [15] in which Manaus had an estimated prevalence of 78% in November 2020. On the other hand, the obtained relative risk of reinfection is higher than reported in the literature for non-Gamma variants [16–18], especially if we assume that part of the donors classified as probable and possible reinfections were reinfected. The protection conferred by previous infection estimated from a cohort of healthcare workers in United Kingdom [17] that were submitted to regular PCR tests was 84%, similar to the protection of 80.5% obtained from a cohort of confirmed COVID-19 cases in Denmark [16]. In a cohort of confirmed cases in Italy [18], only 0.31% of the previously positive individuals were reinfected, compared to 3.9% of primary infections for the negative cohort. However, confirmed cases are biased towards symptomatic individuals, which are likely to have a smaller reinfection risk than asymptomatic or oligosymptomatic individuals. Also, because the sensitivity of serological assays depends on the disease severity [19], traditional methods may not detect reinfections following a mild infection.

The main limitation of this study is that donors were not sampled frequently enough to robustly detect cases of reinfection, leading to the possible existence of undetected reinfections. We attempted to resolve this issue by classifying the degree of evidence and identifying probable and possible reinfections. Further, repeat negative donors may not represent truly unexposed individuals, since not all PCR + individuals produce antibodies to nucleocapsid proteins [20] and because sparse sampling may have resulted in missing the positive interval. Another limitation is that a small proportion of infections in 2021 were not caused by Gamma, thus our results may be slightly affected by the reinfection rate of the non-Gamma variants.

The clinical relevance of COVID-19 reinfection could not be determined because we did not have access to previous signs and symptom information. Since blood donors are biased towards asymptomatic and mild infections, our reinfection rates cannot be extrapolated to persons with more severe primary disease. Because this work uses a serological definition of infection, the protection against symptomatic or severe infection given by symptomatic or severe primary infection is likely higher than our estimates. For the same reason, this work does not assess the protection given by previous infection against symptomatic reinfection, hospitalization or death, nor the protection conferred by symptomatic or severe infection. Finally, even though our results indicate that immunity acquired by natural infection has a limited duration, we do not assess the transmissibility of reinfections. Reinfections may drive a new surge in regions with high seroprevalence even if previous infection reduces transmissibility, but in this case a larger incidence of reinfections is necessary to produce a new outbreak.

## Conclusions

Our data suggest that reinfection by Gamma is common and more frequent than has been detected by traditional approaches. The estimated reinfection rates suggest that the Gamma variant may induce a higher reinfection risk than previous non-Gamma variants, even though the clinical relevance and transmissibility of reinfections was not assessed. Because most blood donors had oligosymptomatic or asymptomatic infections, the obtained protection against reinfection does not extend to cohorts containing only hospitalized or symptomatic individuals. Overall, our results reinforce concerns over the risk of reinfection particularly as variants continue to evolve, and demonstrate that repeat blood donor serosurveillance is valuable for documenting rates and correlates of reinfection that complement surveillance for reinfections or vaccine breakthrough infections based on serial swab-based surveillance programs.

## Supplementary Information

**Additional file 1**: **Appendix**. Materials and Methods. **Fig. S1**. Flowchart describing how repeat blood donors were selected and classified. **Fig. S2**. Serial results obtained with the anti-N assay for each group of donors. **Fig. S3** Serial results obtained with the anti-S assay for each group of donors. **Fig. S4**. Validation of the noise level of the SARS-Cov-2 anti-N IgG chemiluminescence microparticle assay by testing 200 samples in replicate. **Fig. S5**. Racial distribution of repeat blood donors in Manaus.

## Data Availability

Anonymized individual-level data of the 238 selected blood donors along with a data dictionary is available at https://github.com/carlosprete/reinfection_manaus. Data related to all repeat blood donors can be shared upon request.
