# Supplementary material for: Reinfection by the SARS-CoV-2 Gamma variant in blood donors in Manaus, Brazil
Source: BMC Infect Dis. 2022 Feb 5;22:127. doi: 10.1186/s12879-022-07094-y (PMC8817641; doi:10.1186/s12879-022-07094-y)
Supplement: Supplementary file 1 — Additional file 1: Appendix. Materials and Methods. Fig. S1. Flowchart describing how repeat blood donors were selected and classified. Fig. S2. Serial results obtained with the anti-N assay for each group of donors. Fig. S3 Serial results obtained with the anti-S assay for each group of donors. Fig. S4. Validation of the noise level of the SARS-Cov-2 anti-N IgG chemiluminescence microparticle assay by testing 200 samples in replicate. Fig. S5. Racial distribution of repeat blood donors in Manaus. [file 12879_2022_7094_MOESM1_ESM.pdf]

Reinfection by the SARS-CoV-2 Gamma variant in blood donors in Manaus, Brazil

Supplementary Appendix

Table of Contents

**Contents**	**Page**
Materials and Methods	1
Supplemental Figure 1 – Flowchart describing how repeat blood donors were selected and classified.	3
Supplemental Figure 2 – Serial results obtained with the anti-N assay for each group of donors.	4
Supplemental Figure 3 – Serial results obtained with the anti-S assay for each group of donors.	4
Supplemental Figure 4 – Validation of the noise level of the SARS-Cov-2 anti-N IgG chemiluminescence microparticle assay by testing 200 samples in replicate.	5
Supplemental Figure 5 – Racial distribution of repeat blood donors in Manaus.	6
References	6

Materials and Methods

1. Definition of the groups of donors

From all 3,655 repeat blood donors tested with the anti-N assay, we selected only donors with three or more donations because it is not possible to infer reinfection based on two time points. We also required donors to have one donation between March 1st, 2020 and June 30th, 2020, and one donation after January 1st, 2021. This is because most infections in the first wave happened between March and June, thus this requirement helps avoiding selecting donors that had their first sample collected many months after the date of infection, which may have a false negative result if they have already seroreverted when their first sample was collected. If this requirement is not employed, some cases of reinfection may be misclassified as infection by Gamma because the first infection was not detected. It is worth noting that this requirement depends only on the date of donation, and does not add a bias towards positive or negative results.

240 donors met these criteria and were tested with the anti-N assay. We excluded two donors who had their first positive anti-N result in November or December 2020 (when the prevalence of Gamma was small, but rising) because it is not possible to determine if they were infected by Gamma or a non-Gamma variant. The samples from the 238 selected donors were retested with the anti-S assay, except for 18 samples that did not have enough volume to be retested, causing 15 donors to be classified as "Unknown" for the anti-S assay.

The 238 and 223 selected donors for the anti-N and anti-S assays respectively were divided into five groups for each assay, and these groups were then combined according to **Table 1** to obtain the final classification for each donor. The definition of these groups depends on a predefined parameter $\Delta t_{\min}$ used to define the expected behavior of non-reinfected individuals. This parameter represents the minimum interval between donations necessary to accept a probable reinfection, and it is estimated based on donations that occurred before the incidence of Gamma became significant (i.e., donations up to and including October 2020).

The objective of defining $\Delta t_{\min}$ is to avoid misclassifying donors as reinfected when samples were collected during the seroconversion period – that is, we consider that $\Delta t_{\min}$ is much greater than the period of seroconversion. Before estimating these parameters, we added to all donors an artificial negative donation with CIMA result 0·01 S/C on February 28, 2020, before the beginning of the epidemic in Manaus. This is because at that date SARS-CoV-2 had not yet been introduced to the population, which was presumably completely immunologically naïve at that time.

Let $N(\Delta t_{\mathrm{i}})$ be the number of donors that have at least one pair of successive positive results before November 2020 separated by an interval $\Delta t \geq \Delta t_i$. The function $N(\Delta t_i)$ represents the number of possible reinfections observed in 2020, for a given choice of Δt_i. We first estimate $\Delta t_{\min}$ as the smallest interval Δt_i such that $N(\Delta t_i) = 0$, obtaining $\Delta t_{\min} = 141$ days and 126 days for the anti-N and anti-S assays respectively. It is worth noting that changing the value of $\Delta t_{\min}$ does not substantially change the number of probable reinfections because all cases of probable reinfections have samples separated by a large interval.

2. Assessing the measurement error of the SARS-Cov-2 anti-N IgG chemiluminescence microparticle assay

We define the CIMA test to be positive if the measured signal-to-cutoff (S/C) is higher or equal to 0·49 for the anti-N assay. This is the lowest value of range defined by the manufacturer (CIMA, Abbott Park, IL, USA) and provides a specificity of 97·6% (95% CI 96·3% - 98·5%) based on 20 false-positives in 821 pre-pandemic blood donation samples in Manaus, and a peak sensitivity (prior to waning) of 91·7% (95% CI 87·0 – 94·4) based on 177 positive samples out of 193 PCR-positive symptomatic convalescent plasma donors tested 20-50 days following symptom onset[1]. For the anti-S assay, we use the cutoff of 50·0 recommended by the manufacturer to determine if the test is positive. In our analyses, we do not apply any correction based on the sensitivity and specificity of the assay.

Even though the anti-N assay has high sensitivity and specificity, it produces results that are subject to measurement error, which results in variation in S/C that does not reflect a biological change, but is simply variation within the limit of precision of the test. If this variation is not small, sequential donations may have a V-shaped curve even if reinfection has not occurred, leading to an overestimation of the reinfection rate. To assess the amount of measurement error, we tested 200 samples in replicate from blood donors that donated in February 2021 in São Paulo.

Supplemental Figure 4 shows the measured S/C for the first and the second test of each sample. The absolute deviation of each pair of measured S/C had a median of 0·00 and a 95% confidence interval of $0.00 - 0.09$. If only positive results were considered, the median deviation increases to 0·02 (95% CI 0·00 - 0·16), and the relative deviation obtained by dividing the absolute deviation by the first result has median 1·21% (95% CI 0·00% - 7·3%) for positive results.

Therefore, the assay employed in this study yields results with a small amount of measurement error. For this reason, a sequence of serial samples is unlikely to be misclassified as a case of reinfection due to measurement noise.

The measurement error was not assessed for the anti-S assay, but this does not affect the robustness of our results because we used the anti-S assay as a secondary validation of the results obtained with the anti-N assay. Nevertheless, the data presented by Germanio et al[2] shows that the S/C measured with the anti-S assay consistently wanes over time, suggesting a small noise level as well.

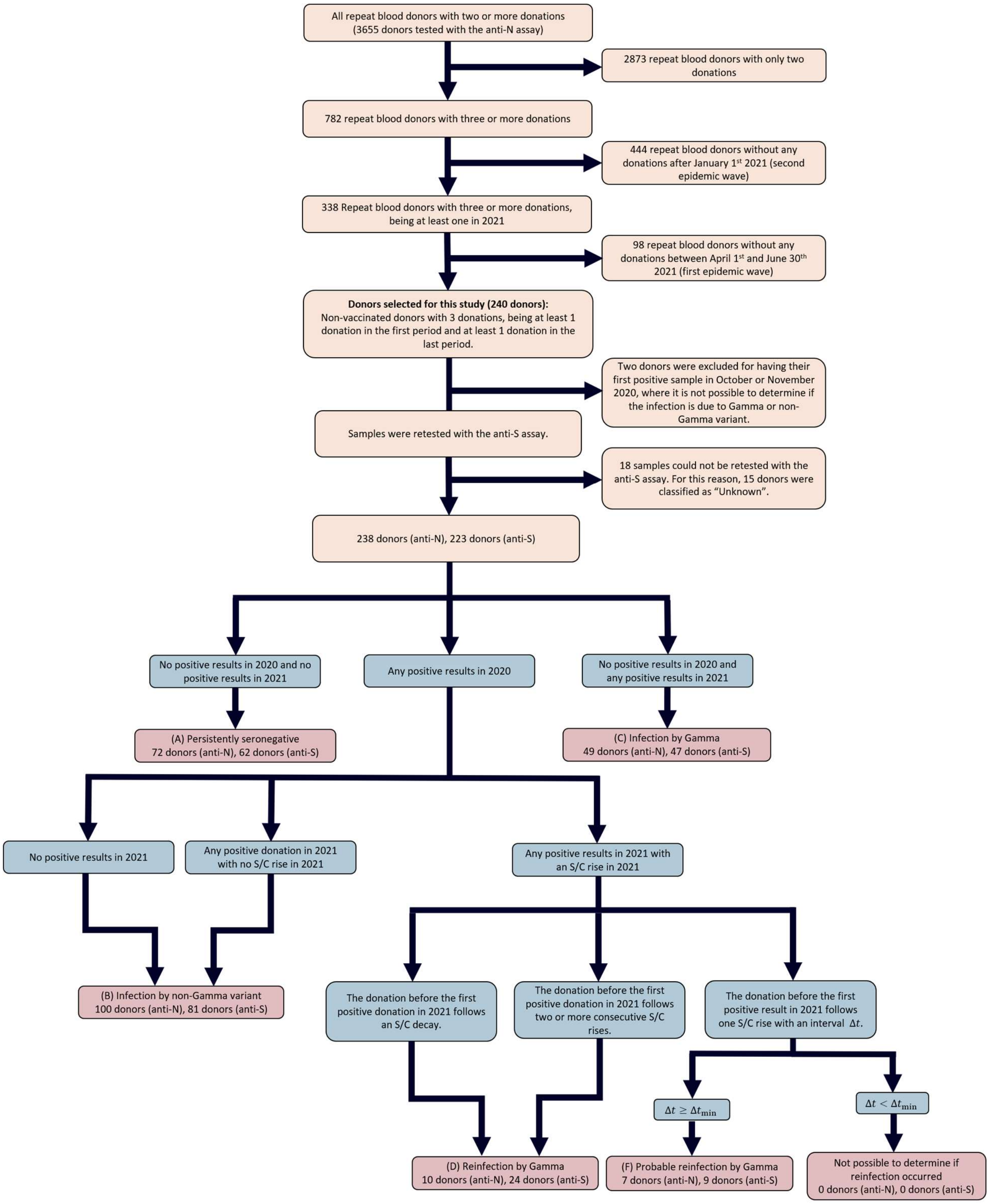

Supplemental Figure 1 – Flowchart describing how repeat blood donors were classified into the groups shown in Figure 1. We used $\Delta t_{\min} = 141$ days and $\Delta t_{\min} = 126$ days for the anti-N and anti-S assays respectively.

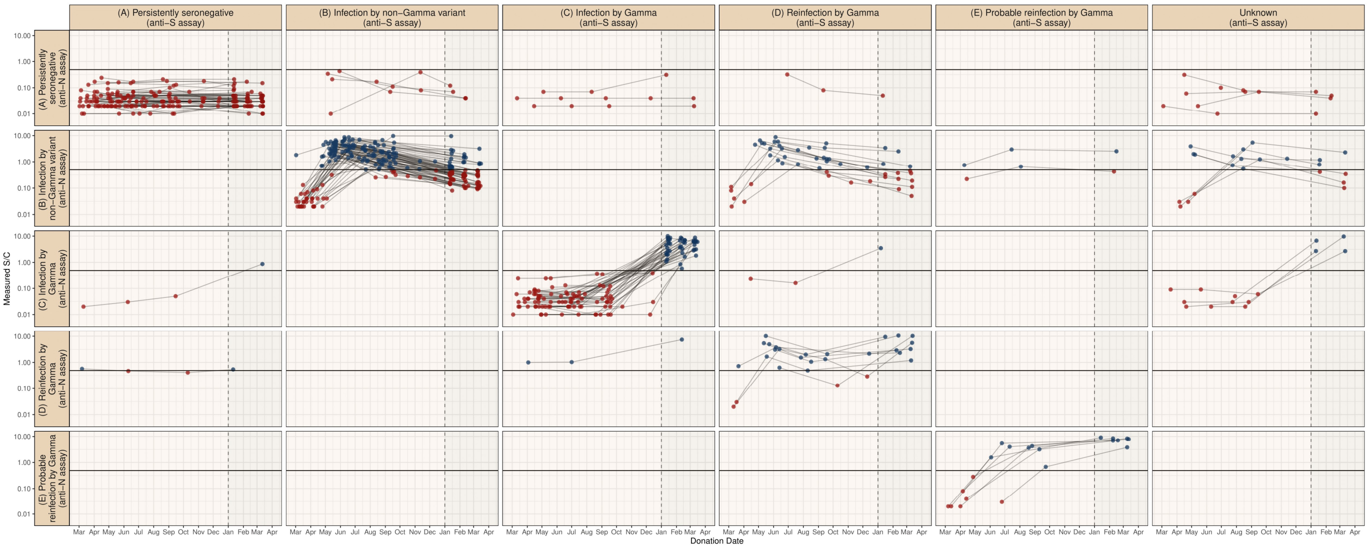

Supplemental Figure 2 – Serial results obtained with the anti-N assay for each assay-specific group of donors.

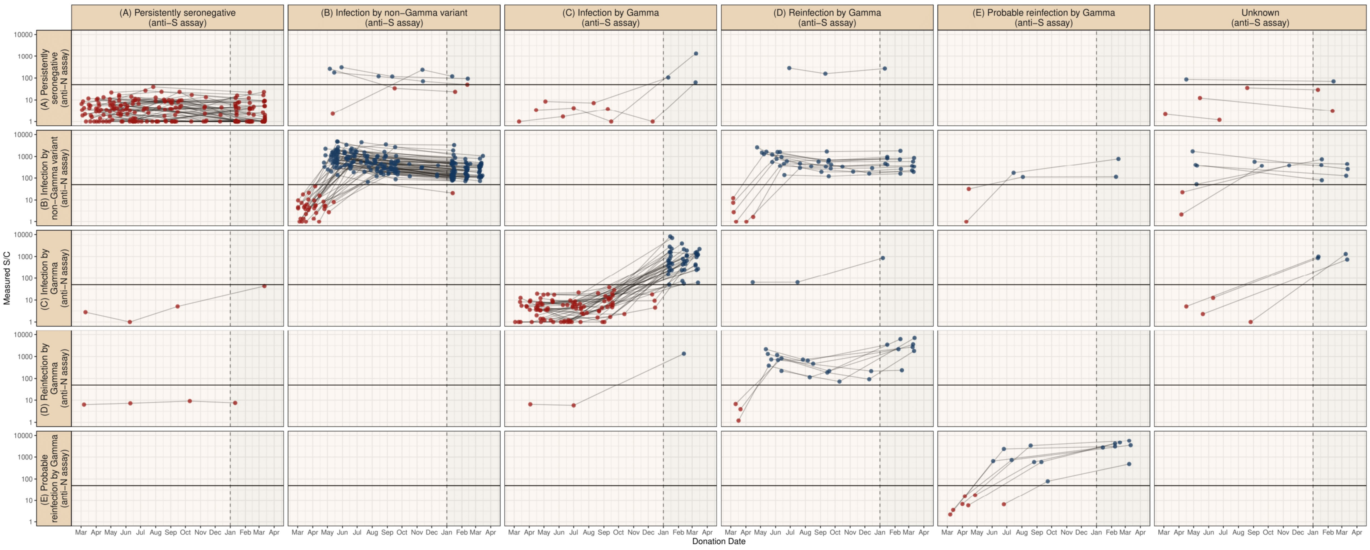

Supplemental Figure 3 – Serial results obtained with the anti-S assay for each assay-specific group of donors.

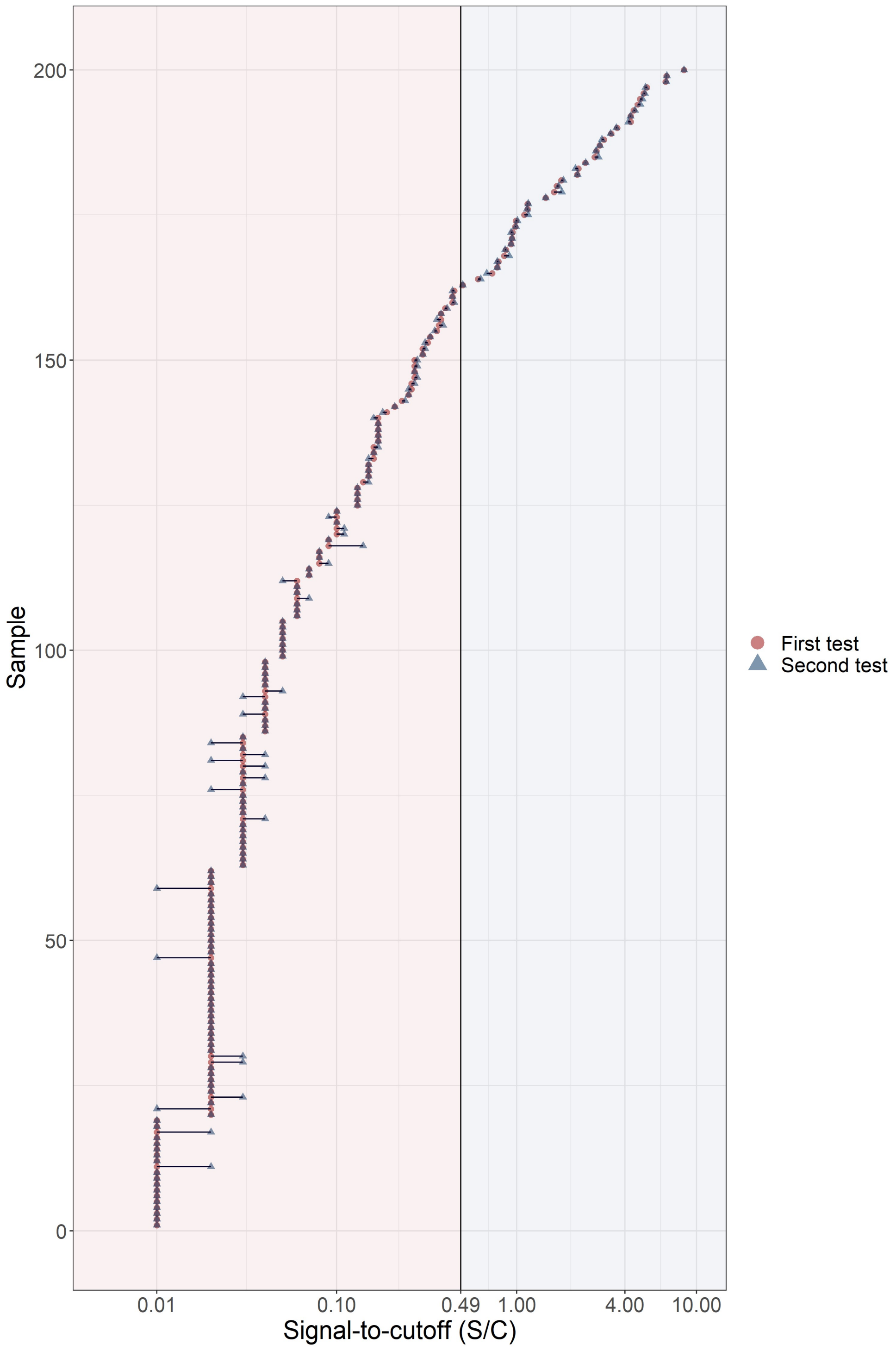

Supplemental Figure 4 – Validation of the noise level of the SARS-Cov-2 anti-N IgG chemiluminescence microparticle assay by testing 200 samples in replicate. Results corresponding to the same sample are connected by a horizontal line. The assay produces consistent results with very little variation.

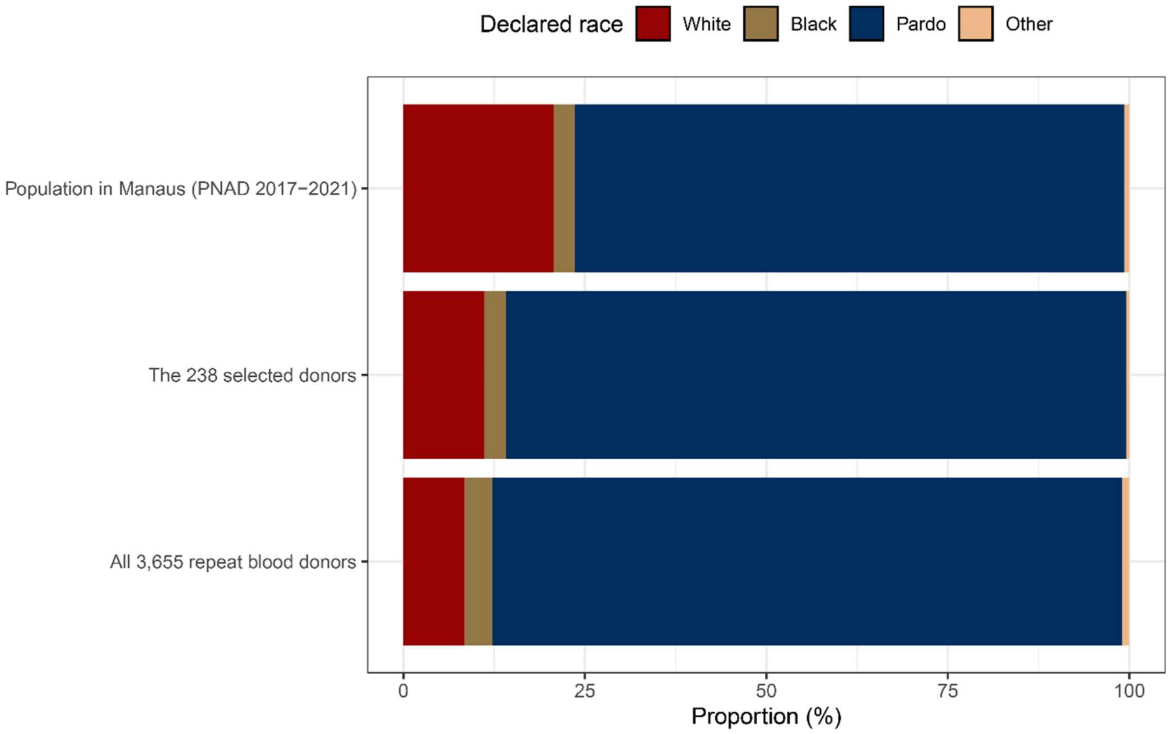

Supplemental Figure 5 – Comparison of the racial distribution of all 3,655 repeat blood donors with two or more donations, the 238 selected donors and the racial distribution of the population Manaus estimated from the quarterly household survey PNAD-Contínua (available at https://sidra.ibge.gov.br/Tabela/6403) using samples from 2017 to 2021.

References

1. Buss LF, Prete CA, Abrahim CMM, Mendrone A, Salomon T, De Almeida-Neto C, et al. Three-quarters attack rate of SARS-CoV-2 in the Brazilian Amazon during a largely unmitigated epidemic. Science (80-). 2021;371:288–92. doi:10.1126/science.abe9728.

2. Germanio C Di, Simmons G, Kelly K, Martinelli R, Darst O, Azimpouran M, et al. SARS-CoV-2 antibody persistence in COVID-19 convalescent plasma donors: Dependency on assay format and applicability to serosurveillance. Transfusion. 2021. doi:10.1111/TRF.16555.
